# The Rod of Health

**Published:** 1887-09-10

**Authors:** 


### The Rod of Health.

By Bookworm.

Since the days of Solomon, and owing in great part to the solemnly pronounced dictum of that sage, there has been a devout belief cherished by parents and guardians, though not shared by children and wards, that the rod is a fine moral tonic. The wise king's proverb has been crystallised into a rhyme so ancient that the date of its composition is lost in the mists of antiquity, though it is still well known in the nurseries of to-day—

"Solomon said in accents mild,
Spare the rod and spoil the child;
Be they man or be they maid,
Whip them and wallop them, Solomon said."

But though parents, schoolmasters, and persons in authority generally have always shown a sincere and practical faith in the good effects of flagellation on the intellect and conscience, it was reserved for a German philosopher to evolve the grand truth that the rod is an excellent medicine for the body as well as for the soul.

This great man (to whom posterity has not done the justice he deserves) was Dr. Christian Franz Paullini, who in 1608 published, at Frankfort-on-Maine, the treatise entitled *Flagellum Salutis*—"The Rod of Health." In it he proves that all the ills that flesh is heir to may be cured, or at least ameliorated, by the use of the rod—birch, cane, or cat-o'-nine-tails, *ad libitum*. It is true that he does not differentiate as accurately as the ordinary mind might desire between physical and mental diseases; but in his habit of regarding all ailments as due to bodily causes, and curable by corporal remedies, his views are quite as advanced as those of the most noted physiologist of our own day. Spencer or Darwin might accept him as a brother, or even as a master, for his ideas on the physical origin of moral defects go far beyond theirs.

Dr. Paullini declares the rod to be a universal specific, and goes on to explain its action on the human frame. It "stirs up the stagnating juices, dissolves the precipitated salts, purifies the coagulating humours of the body, clears the brain, circulates the blood, and braces the nerves"; in short, contains within itself the therapeutic qualities of every drug known to the Pharmacopœia. The most widely advertised nostrum of modern days cannot be compared with it in all-round usefulness.

Lest, however, the sceptical mind should cherish any doubts as to the universal value of the remedy, the doctor is careful to substantiate the claims of the rod to medicinal virtues by narrating instances of the cures wrought by it, drawn chiefly from his own experience.

For melancholy the rod is an infallible cure, and should always be used with the greatest promptitude; for melancholy is usually caused by love, and love results eventually in idiocy or insanity. (Remember, dear young lady readers, that this is Paullini's opinion, not our own.) "I saw," he says, "an instance of the good effect of this treatment at Amsterdam. A youth there, the son of an artisan, fell in love with the mayor's daughter. He could neither eat nor sleep, nor indeed do anything rational, so absorbed was he by his passion. His father, not suspecting the cause of this depression of spirits, called in a physician, who plied him with all the drugs in his laboratory, without producing any salutary effect." At last a letter from the Dutch Juliet to her Romeo fell into the worthy artisan's hands. He at once comprehended his son's case, and took the remedy into his own hands. He sent the love-lorn youth to the public whipping-place, with certain instructions. . . . The result was all that could be desired. The youth returned perfectly cured of his infatuation, and that little romance came to an untimely end.

Who can tell from what sad fate this prompt and vigorous treatment saved the young man! He might have shared the hapless fate of that youth who died of a broken heart, at the post-mortem examination of whose body it was found that the heart was shrivelled (absolute rupture is not recorded), the liver shrunk away to nothing, the lungs corroded, and the skull entirely void of any trace of brains!

The rod is the grand remedy for all childish complaints. For the causeless crying in which children often indulge a homœopathic treatment is recommended—*similia similibus*, giving them something to cry for, as the nurses say. We have noticed that children always seize with avidity on the excuse thus given them, and howl twice as lustily as before.

The treatment specially recommended for deafness is a sound box on the ear. This is entirely contrary to the ideas of modern doctors, who tell of cases in which a sudden blow on the ear has broken the tympanum and caused incurable deafness. But Dr. Paullini's treatment is still both popular and successful in cases where children fail to pay heed to the commands of their parents on the plea of "not having heard" them. He tells the story of a deaf youth who was destined for the ministry; but, disliking the prospect, ran away and apprenticed himself to a tailor. On his parents discovering what he had done they applied Dr. Paullini's remedy, with such success that both his deafness and his tailoring propensities were perfectly cured, and he became a highly respected Lutheran pastor. To the end of his days, however, he retained a talent for mending his clothes; which, if his living was not a very rich one, might be looked upon as rather a fortunate *sequela* of his youthful illness.

The best cure for blindness, entire or partial, is a sudden blow on the head. This discovery, it appears from Dr. Paullini's illustrative anecdotes, was made by accident. An old man, who had all his life suffered from short sight, one day mounted his horse and set out for market. On the way the horse stumbled and threw his rider, who landed head foremost on a stone. The stone pierced his skull, and by the aperture thus made the dense vapours which had so long obscured his sight were permitted to escape; and from that hour till the day of his death the old gentleman's keenness of vision was the envy of his friends, when it was not their terror.

In all the foregoing examples there is the possibility of a doubt arising in the modern mind as to the necessary connection between the remedy applied and the cure effected; but the treatment suggested by our author in cases of dislocation of the jaw commends itself to the practical intelligence of the nineteenth century. A sudden blow on the cheek is the method still occasionally employed to cure this painful though rather grotesque accident. On this subject listen to the following story:—

The oriental traveller, Nicholas Vorburg, chanced to visit Agra, the capital of the great Cham. The Eastern potentate was graciously pleased to welcome the European with effusion; and asked him to share the royal dinner, which was on the point of being served. Among other viands, a bowl of rice was brought in; and the great Cham, who had just returned from hunting, and was ferociously hungry, plunged his hands into it, and thus, with regal grace and simplicity, conveyed it to his mouth. But take warning, young friends, who are sometimes guilty of too great haste in eating! He had miscalculated the capacity of his mouth, and, trying to open it wide enough to admit the whole handful of rice, he dislocated his royal jaw. Horror struck the court at this terrible sight. The servants screamed; the nobles gazed helplessly at each other; and priests and sorcerers, confident that they could do no harm, and trusting that they might do some good, commenced a series of incantations, meant to exorcise the evil spirit who was thus, as they thought, distorting their master's countenance; but none offered to help the poor monarch, who, sitting upon his throne with his mouth open, his eyes protruded, and his face purple, seemed in immediate danger of some tremendous catastrophe. In this dilemma Vorburg came to the rescue. To the terror and amazement of the courtiers, he ran up the steps of the throne, and with a sacrilegious hand struck the Emperor a thwacking blow on the cheek. In a moment the jaw had resumed its proper position, and the monarch, though breathless and gasping, was himself again. But the courtiers, indignant at the outrage upon the royal person (the intention of it being quite beyond their comprehension), drew their swords, and were about to reward Vorburg for his timely succour by hacking him in pieces; when the great Cham recovered his breath to a sufficient extent to stay the zeal of his too loyal subjects, and reward his preserver with a present of a thousand rupees. Had the traveller not been there the great Cham might have met with a fate similar to that which befell a certain king of Spain, who was burned to death because none of his attendants dared to take the liberty of throwing his Majesty down and wrapping him in the hearth-rug.

In tertian fever the rod is, Dr. Paullini says, a most successful cure, though the result of the case he quotes scarcely goes to prove his statement.

There was once a lawyer who suffered from this disease, but who, in the intervals of the attacks, was able to attend to his duties in court. In the course of one of his pleadings he had taken occasion to ridicule a neighbouring nobleman. The latter did not forgive his legal adversary, but, like the villains of melodrama, he said, "I will dissemble." He therefore sent a courteous letter to the lawyer, saying that he wished to have a little private conversation with him. The lawyer jumped to the conclusion that his lordship wished to place some business in his hands, and hastened to the castle, the owner of which had ordered that as soon as he entered, all the gates should be shut and fastened securely. Then the nobleman interviewed the lawyer.

"Vile bloodhound of the law!" was the flattering commencement of his speech; then he proceeded to load his victim with all manner of reproaches—in short, to tell him exactly what he thought of him. Finally he unfolded the intention with which he had decoyed him into his power.

"You insulted me publicly," said he, "and I intend to punish your insolence in the manner most humiliating to your pride. I will, however, give you the choice of two methods of expiating your fault. You may either sit on an ant-hill, clad in no other garment than nature has given you, until you have learned by heart the seven penitential psalms; or if you prefer it, you may, attired in the same costume, run the gauntlet round my courtyard, where my servants will be placed, each armed with a stout rod."

The unfortunate lawyer cast himself at his tormentor's feet and implored his mercy. He pleaded that he had abused his lordship only in the way of business, and that in private life he had the highest respect for him. The noble replied only by an incredulous grunt.

"Think of my wife and childen," cried the lawyer.

The nobleman put on a bewildered air. "What have they to do with it?" he asked, politely. "I have not the slightest intention of injuring either the lady or her family. My business is with you alone."

"But I have been ill," said the hapless man of law. "I am scarcely yet recovered from an attack of fever, and either of the punishments you suggest would be the death of me."

His lordship considered this view of the matter for a few moments. At last he answered, "That is a point which can be settled only by experiment. For my own part, I believe that the ants or whips will produce a counter-irritation which may be beneficial. Still, I may be wrong. Should the experiment terminate as you think, I will candidly own my mistake. And now oblige me by making your decision."

With many groans the miserable creature came to the conclusion that running the gauntlet would not take so long to get over as the penitential psalms accompanied by ants. So he chose the serving-men's whips.

A mass of cuts and bruises, he returned to his family He never suffered from fever nor from any other ailment again. So far the nobleman was right. Let us hope that he dealt generously with the widow.

It is always interesting to learn something of the lives of men of note, but of the author of *Flagellum Salutis* nothing seems to be known. We are given no information about the duration of Dr. Paullini's life, nor of the date and circumstances of his death. We have, however, a theory of our own about the latter event. Our surmise is that he was set upon and flogged to death by a mob of infuriated school-boys, whose instructors had displayed too fervent a belief in the merits of the doctor's discovery; and who were therefore anxious to show him that it was possible to have too much of even such a good thing as the "Rod of Health."

